# The Role of Radiation Therapy in the Management of Metastatic Melanoma in the Brain

**DOI:** 10.1155/2012/294735

**Published:** 2012-04-11

**Authors:** Angela Hong, Gerald Fogarty, Michael A. Izard

**Affiliations:** ^1^Melanoma Institute of Australia, Poche Centre, North Sydney, NSW 2060, Australia; ^2^Sydney Medical School, The University of Sydney, Sydney, NSW 2006, Australia; ^3^Department of Radiation Oncology, Royal Prince Alfred Hospital, Sydney, NSW 2050, Australia; ^4^Mater Sydney Radiation Oncology, Radiation Oncology Associates, and Genesis Cancer Care Pty Ltd, Sydney, NSW 2060, Australia; ^5^Australian School of Advanced Medicine, Macquarie University, Sydney, NSW 2109, Australia

## Abstract

Brain metastasis is common in patients with melanoma and represents a significant cause of morbidity and mortality. There have been no specific randomized trials for patients with melanoma brain metastasis, so treatment is based on management of brain metastasis in general and requires multidisciplinary expertise including radiation oncology, neurosurgery, medical oncology, and palliative care. In this paper, we summarize the prognosis, general management, and the role of radiation therapy in the management of metastatic melanoma in the brain.

## 1. Introduction

Metastatic melanoma in the brain is a serious event in patients with melanoma because of the poor prognosis and potential impact on quality of life. Symptomatic metastases represent the initial site of metastatic spread in 20% but may occur at any time during the course of the disease [1]. Autopsy data have shown that up to 75% of patients who died from metastatic melanoma had brain metastases [2, 3]. Two large institutional series of 686 and 702 patients [3, 4] indicate a generally poor outcome, with the majority (up to 95%) dying directly from brain metastases. The median survival of patients with multiple metastases was approximately 3-4 months. There were some differences in survival according to treatment received, being 8.9 months for surgery plus whole brain radiotherapy (WBRT), 8.7 months for surgery alone, 3.4 months for WBRT alone, and 2.1 months for supportive care. These differences are a probable reflection of patient selection based on the number of cerebral metastasis, performance status, and extent of extracranial metastasis.

## 2. Prognostic Factors for Survival

The Radiation Therapy Oncology Group Recursive Partitioning Analysis (RPA) Classes have been validated in melanoma [5]. Age (>65 year old) and the number of neurological symptoms (weakness and fatigue) are associated with poorer survival [6]. Ulceration and location on the head and neck region are two main primary tumour characteristics that are associated with poorer survival [6]. The number of cerebral metastases is a significant prognostic factor with better prognosis seen in single or oligometastatic disease (2-3 cerebral metastases). Patients with more than 3 metastases had a median survival of 3.5 months compared with 5.9 months for those with 3 or less metastases (*p* = 0.005). More recently, there is debate on whether it is the number of metastases or the overall intracranial tumour volume that is the relevant factor [7]. The worst outcome is seen in patients with leptomeningeal disease [8]. In all large cohorts of patients with melanoma brain metastases, the absence of extracranial disease was a positive prognostic factor.

MD Anderson Cancer Center analysed the outcomes of 743 patients with metastatic melanoma in the brain treated between 1986 and 2004 [9]. On multivariate analysis, the date of diagnosis was a prognostic factor. The median survival for patients diagnosed before 1996 was 4.14 months compared with 5.92 months for patients diagnosed in 1996 or later (HR 0.75 95% CI 0.59–0.95, *p* = 0.02). The increased use of MRI as a screening tool for brain metastases over time may have contributed to this improvement. In addition, earlier diagnosis of patients with lower burden, asymptomatic brain metastasis might allow for more frequent use of locally directed treatment such as stereotactic radiosurgery or surgical excision. A similar study of patients from the Memorial Sloan Kettering Cancer Center noted that age >65, presence of extracranial metastases, presence of neurologic symptoms and four or more metastases are predictors for poorer survival, although some of these features are self-predicting in that more aggressive treatment options are less likely to be offered [10].

## 3. General Management

The management of metastatic melanoma in the brain depends on the combination of patient, tumor, and treatment factors. The dominant factor determining management has been the number of cerebral metastases. With the wider availability of stereotactic radiosurgery facilities enabling the effective treatment of multiple metastases in a single treatment session, the absolute number of cerebral metastases is now less important than previously. Reports increasingly suggest that the use of stereotactic radiosurgery to treat multiple metastases may have merit, particularly if there are less than 10 lesions, all under 3 cm in size and with limited oedema or mass effect [11–13]. Recent data suggested that the total volume of the metastatic lesions rather than the number of metastases was the limiting factor for radiosurgery technique [7].

**Figure 1:**
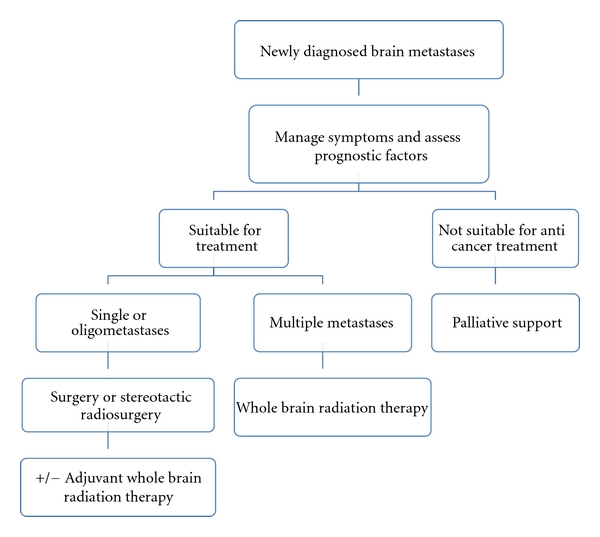
Approaches for management of melanoma brain metastasis.

Our general approach in the management of melanoma metastases in the brain is shown in Figure 1. For patients with a single or oligometastases, the management depends on the performance status, neurological status, the characteristic of the metastases (number, size, and location), and the extent of the extracranial disease. Patients in the RPA class 3 are managed with steroids in conjunction with WBRT. Those with more favourable characteristics should be considered for more aggressive local treatment of the individual metastasis. The two options of local treatment are surgical excision and stereotactic radiosurgery. Surgery has a role in confirming the diagnosis especially as there is no clear relationship between the primary melanoma and the development of brain metastasis. In addition, surgical resection can also provide quick relief in symptoms associated with disease such as shunting in hydrocephalus. The relative advantages and disadvantages of each technique are summarised in Table 1.

## 4. Multiple Metastases

Patients with multiple metastases are generally not suitable for aggressive local treatment. The prognosis is poor, with the majority succumbing to progressive intracranial metastases within months, irrespective of treatment. Application of the RTOG recursive partitioning analysis to 74 patients with cerebral metastases from melanoma produced median survival of 10, 6, and 2 months, respectively, for RPA Classes I–III, respectively, with a median survival of 5.5 months for the entire group [5].

Initial management includes the use of steroids, typically 4–16 mg of dexamethasone per day. This usually results in rapid symptomatic, but often short-term, improvement in approximately 50% of patients. Whole brain radiation therapy may produce a small survival advantage compared with steroids alone and may allow reduction in the steroid dose. In addition to whole brain radiation therapy, surgical removal, or stereotactic radiosurgery of a dominant and symptomatic lesion should be considered. Conversely, patients with a poor performance status who have not responded to steroids may be better treated with supportive care.

The technique of whole brain radiation therapy is a pair of parallel opposed lateral 6 MV photon fields. Commonly used regimens are 20 Gy in 5 fractions and 30 Gy in 10 fractions. For good performance patients with minimal extracranial disease, there might be an advantages for higher dose of whole brain radiation therapy based on a retrospective study [14]. Rades et al. compared the outcomes of 33 patients treated with 30 Gy in 10 fractions with 18 patients treated with higher doses (40 Gy in 20 fractions or 45 Gy in 15 fractions). In the multivariate analysis, higher doses (*p* = 0.010), less than four brain metastases (*p* = 0.012), no extracranial metastases (*p* = 0.006), and RPA class 1 (*p* = 0.005) were associated with improved overall survival.

In an attempt to improve survival of patients with multiple brain metastases, radiation has been combined with a variety of chemotherapy agents, including temozolomide, thalidomide, and fotemustine without much success [15–18]. The most recent study is a phase 2 study combining whole brain radiation therapy (30 Gy in 10 fractions) with temozolomide and thalidomide in 39 patients [16]. The response rate was 7.6% and a median time to progression of 7 weeks.

## 5. The Role of Stereotactic Radiosurgery in Single or Oligometastases

The term radiosurgery was originally coined by Lars Leksell to describe the use of a multisource cobalt system (Gamma Knife) to deliver radiation to a defined target using stereotactic principle. It aims to deliver an ablative dose to the target while limiting the dose to surrounding normal tissue. The latest version of the Gamma Knife (Perfexion) uses 192 cobalt sources arranged circumferentially in a noncoplanar fashion, permitting smaller doses to the surrounding normal brain tissue and a lower integral dose. The associated improvements in planning software and design modifications have resulted in the ability to treat multiple targets in one session [19]. Historically, this has relied on frame-based stereotactic approaches that can accurately localise the tumour and target the beam in three-dimensional space. Options now include frameless image-guided approaches such as fixed beam intensity-modulated radiotherapy, helically delivered intensity-modulated radiotherapy (TomoTherapy), and image-guided robotic radiosurgery (Cyberknife). Arc-based intensity-modulated radiotherapy techniques (VMAT and RapidArc) can also achieve highly conformal image-guided treatment in very short treatment times [20]. Linear accelerator-based stereotactic radiosurgery will use a limited number of fields, usually (but not always) in a coplanar fashion.

The dose of radiosurgery depends on the size of the target lesion and the location. The RTOG 90-05 study was designed to determine the maximum tolerable dose of radiosurgery in patients with recurrent previously irradiated brain metastases (excluding lesions in the brain stem) [21]. The maximum tolerable doses of single fraction radiosurgery for patients with recurrent previously irradiated brain metastases were 24 Gy, 18 Gy, and 15 Gy for tumors ≤20 mm, 21–30 mm, and 31–40 mm in maximum diameter, respectively. In the multivariate analysis, those who were treated on a linear accelerator (versus the Gamma Knife) had a 2.84 greater risk of local progression. However there are no randomised data showing clear superiority of any one stereotactic radiosurgery system.

Mathieu et al. from the University of Pittsburgh reviewed the experience of 244 patients with 754 melanoma metastases treated with Gamma Knife radiosurgery without adjuvant whole brain radiation therapy [22]. Local control was achieved in 86.2% of the metastases. Overall, 54 patients (30.9%) had progression of at least one metastasis after radiosurgery. The median time to progression was 2.9 months. Fifty-one patients (24.8%) underwent whole brain radiation therapy after radiosurgery because of the development of multiple new brain lesions. Multiple lesions and failure to provide systemic immunotherapy were predictors for the occurrence of new brain metastases, which developed in 41.7% of the patients. Progressive cerebral disease was the cause of death in 40.5% of the patients. Corticosteroids were not needed or were discontinued after radiosurgery in 52.4% of the patients. On multivariate analysis, the use of whole brain radiation therapy was not a factor that influenced local control or distant intracranial control (*p* = 0.061). A more recent update of the University of Pittsburgh's experience on 333 consecutive patients with 1570 metastatic melanoma lesions treated with Gamma Knife radiosurgery showed the long-term local control rate was 73% and the actuarial survival rates were 70% at 3 months, 47% at 6 months, 25% at 12 months, and 10% at 24 months [23]. About 25% of 259 patients who had followup imaging after stereotactic radiosurgery had evidence of delayed intratumoral haemorrhage. Factors associated with longer survival included controlled extracranial disease, better performance status, fewer number of brain metastases, no prior use of whole brain radiation therapy or chemotherapy, treatment with immunotherapy, and no intratumoral hemorrhage before radiosurgery.

The potential morbidity of stereotactic radiosurgery includes progression or worsening of cerebral oedema symptomatic in 4–6% of patients within 1-2 weeks of treatment, seizures within 1-2 days in 2–6%, and delayed radiation necrosis in 2–11% [21, 24, 25]. This risk increases with prior treatment, larger volumes treated (both larger lesions and larger numbers of lesions), and larger doses delivered.

## 6. The Role of Whole Brain Radiation Therapy in Melanoma after Local Treatment of Metastases

The role for whole brain radiation therapy after surgery or stereotactic radiosurgery of the single or oligometastasis is controversial and there is no level 1 evidence in this scenario. The rationale of whole brain radiation therapy is to treat microscopic disease at the site of initial metastasis and elsewhere in the brain to maintain long-term cerebral control. Adjuvant systemic therapy is generally not used as the brain is considered a sanctuary site for chemotherapy although this assumption has been recently challenged by responses in the patients with B-Raf mutant melanoma treated with B-Raf inhibitor [26]. However opponents of whole brain radiation therapy argue that melanoma is radioresistant and that whole brain radiation therapy can potentially cause late neurocognitive deficits. The Australia and New Zealand Melanoma Trials Group (ANZMTG) and Trans-Tasman Radiation Oncology Group (TROG) are conducting a phase 3 randomised trial to address the role of whole brain radiation therapy after local treatment of 1 to 3 melanoma metastases [27]. Eligible patients are randomised to whole brain radiation therapy or observation after their local treatment of the brain metastasis. The primary end point of the trial is distant intracranial control. This trial also includes detailed neurocognitive and quality of life assessments. Until this trial is reported, clinicians will have to rely on data from other randomised trials included patients with metastatic disease from all histologies.

Several randomised studies including metastasis from all histologies have provided good evidence for the use of whole brain radiation therapy after local treatment of oligometastases in terms of an improvement in intracranial control. The number of patients with melanoma in these trials is typically small. Aoyama et al. compared whole brain radiation therapy and stereotactic radiosurgery (65 patients) to stereotactic radiosurgery alone in patients with 1–4 brain metastases of any histology [28]. Over 65% of the patients had metastatic lung cancer, while the number of melanoma patients per arm was not mentioned. The whole brain radiation therapy dose was 30 Gy in 10 fractions over 2 weeks. There was no difference between the two groups with respect to overall survival, neurological toxicity, neurological functional preservation, and neurological death. The median survival time was 7.5 months with whole brain radiation therapy plus stereotactic radiosurgery compared to 8 months with stereotactic radiosurgery alone. The 12-month actuarial brain tumour local recurrence rate was 46.8% in the whole brain radiation therapy plus stereotactic radiosurgery group and 76.4% in the stereotactic radiosurgery alone group (*p* = 0.001). Fifty-five patients had new brain metastases at distant sites (21 in the whole brain radiation therapy plus stereotactic radiosurgery group and 34 in the stereotactic radiosurgery-alone group). The 12-month actuarial rate of developing distant brain metastases was 41.5% in the whole brain radiation therapy plus stereotactic radiosurgery group and 63.7% in the stereotactic radiosurgery-alone group (*p* = 0.003). The univariate analysis showed that patients with 2–4 metastases had a higher risk for developing distant intracranial disease than those with single metastasis (*p* = 0.03), but this did not reach significance on multivariate analysis (*p* = 0.06).

More recently, EORTC reported a randomized trial of 359 patients with 1 to 3 brain metastases from all solid tumours randomised to either observation or whole brain radiation therapy of 30 Gy in 10 fractions after local treatment (surgery or stereotactic radiosurgery) [29]. The majority (53%) of patients had primary lung cancer and only 5% had metastatic melanoma. After surgery, at 2 years, whole brain radiation therapy significantly reduced the probability of relapse at initial sites from 59% to 27% (*p* = 0.001) and at distant intracranial sites from 42% to 23% (*p* = 0.008). After stereotactic radiosurgery, whole brain radiation therapy reduced the probability of relapse at initial sites from 31% to 19% (*p* = 0.04) and at distant intracranial sites from 48% to 33% (*p* = 0.023) at 2 years. The median progression-free survival was slightly longer in the whole brain radiation therapy arm compared with the observation arm (4.6 months versus 3.4 months; *p* = 0.02) but there was no difference in overall survival between the two arms. Eighty-one percent of the patients had single metastases but there was no analysis of one versus more than one metastases. This trial included neurocognitive and the quality of life assessments which have not yet been reported.

There are also a number of single institution retrospective series in melanoma patients with their inherent selection biases. Fife et al. reviewed the outcomes of 686 patients at the Sydney Melanoma Unit (now the Melanoma Institute Australia) [4]. There was no significant difference in the median survival between the 158 patients treated with surgery and whole brain radiation therapy and the 47 patients treated with surgery alone (8.9 months versus 8.7 months, *p* = 0.21). Sampson et al. also reported no difference in median survival in patients treated with whole brain radiation therapy after surgery or surgery-alone patients (median survival of 9 months, *p* = 0.99). However, patients treated with whole brain radiation therapy were more likely to remain without neurological deficits or experience an improvement (81.7%) after completion of therapy than those who did not (57.7%, *p* = 0.01).

Selek et al. reported on 103 patients with 153 intracranial melanoma metastases treated with stereotactic radiosurgery [30]. Sixty-one patients (59%) had single brain metastasis. Treatment was stereotactic radiosurgery alone (61 patients), stereotactic radiosurgery with whole brain radiation therapy (12 patients), and salvage stereotactic radiosurgery after whole brain radiation therapy (30 patients). The overall incidence of distant brain metastasis-free survival did not differ significantly between the group that received initial stereotactic radiosurgery alone and the group that received stereotactic radiosurgery and whole brain radiation therapy (17.6% versus 0%, *p* = 0.27). However this study did not have the statistical power to detect a difference in distant brain metastasis free survival. The initial number of brain lesions (single versus multiple) was the only factor with a significant effect on distant brain metastasis-free survival at 1 year: 23.5% for single metastases and 0% for multiple lesions (*p* < 0.05). Samlowski et al. performed a retrospective analysis of 44 melanoma patients with five or less brain metastases treated with stereotactic radiosurgery and showed that the addition of whole brain radiation therapy did not improve survival [13]. Buchsbaum et al. reviewed 74 patients with melanoma brain metastases [5]. Survival analysis showed that combined treatment of local and whole brain radiation therapy offered significantly better survival (*p* < 0.0001). The median survival was 8.8 months for the combined therapy group, 4.8 months for the local-therapy-alone group. However, whole brain radiation therapy did not improve the intracranial distant control.

MD Anderson reported a series of patients with solitary melanoma brain metastasis and no extracranial disease [31]. Twenty-two patients received surgical excision and whole brain radiation therapy whole brain radiation therapy and 12 patients were treated with surgery alone. Despite the small sample size, intracranial recurrence rates favoured the combination (5/22 versus 9/12 surgery alone, *p* = 0.01). Median overall survival was 18 months in the combination therapy group versus 6 months for surgery alone (*p* = 0.002). These data argue that whole brain radiation therapy can decrease intracranial progression and may even convey a survival benefit in patients without active extracranial disease as a competing cause of death.

One concern of delivering whole brain radiation therapy after local treatment of oligometastases is the potential neurological deficit. Preclinical and early clinical evidence suggests that a neural stem cell compartment in the hippocampus is central to the pathogenesis of neurocognitive deficits observed after cranial irradiation. Modern intensity-modulated radiotherapy technologies, such as helical tomotherapy and volumetric modulated arc therapy can conformally avoid the hippocampus during whole brain radiation therapy and therefore potentially reduce the risk of neurocognitive deficit. A RTOG review of 371 patients with less than 10 brain metastasis from all histologies showed that only 3% of the metastatic deposit were within the 5 mm region around the hippocampus and none within the hippocampus itself [32]. There is an ongoing clinical trial (RTOG 0933) examining the effect of hippocampal avoidance whole brain radiation therapy technique on the neurocognitive function in patients with brain metastases from all histologies.

## 7. Conclusions

Brain metastasis is a common development in patients with metastatic melanoma. The role of radiation therapy in the management is highly variable due to the natural history of the disease. To provide optimal management of the patient with melanoma, the radiation oncologist is an integral part of the multidisciplinary team. Although there have been no randomized trials especially in patients with melanoma brain metastasis, treatment can be guided by the application of evidence for the treatment of brain metastasis in general. A promising new approach to deliver radiation therapy while sparing the hippocampus will hopefully improve the therapeutic ratio and minimise potential long term toxicity.

## Figures and Tables

**Table 1:** Relative advantage of surgery and stereotactic radiosurgery for single or oligobrain metastases.

	Surgery	Stereotactic radiosurgery
Indications	(i) When histological confirmation is needed. (ii) Single or “dominant” lesion. (iii) Prominent cystic component or necrosis. (iv) Prior radiosurgery treatment failure.	(i) Lesion in eloquent locations. (ii) Oligometastatic disease. (iii) When lesions are associated with mild or no clinical symptoms. (iv) Contraindications to craniotomy (e.g., high anesthesia risk and anticoagulation).

Advantages	(i) Prompt symptom relief e.g., from obstructive hydrocephalus, mass effect; midline shift, intratumoral, or intracerebral bleed. (ii) No size limit.	(i) Outpatient day only procedure. (ii) Concurrent chemotherapy or imminent treatment protocol, especially antiangiogenesis therapy.

Disadvantages	(i) Depends on expertise of surgeon. (ii) Lesion needs to be surgically accessible.	(i) Depends on expertise of radiosurgery team. (ii) Lesion needs to be favourable that is, size <3 cm, solid tumor, with homogenous enhancement and minimal vasogenic edema, no hydrocephalus.
